# Long non-coding RNAs LINC00689 inhibits the apoptosis of human nucleus pulposus cells via miR-3127-5p/ATG7 axis-mediated autophagy

**DOI:** 10.1515/med-2022-0544

**Published:** 2022-11-22

**Authors:** Changsheng Wang, Rongsheng Chen, Xitian Zhu, Xiaobo Zhang

**Affiliations:** Department of Spinal Surgery, First Affiliated Hospital of Fujian Medical University, No. 20, Chazhong Road, Taijiang District, Fuzhou City, Fujian Province, 350005, China; Department of Spinal Surgery, First Affiliated Hospital of Fujian Medical University, Fuzhou City, Fujian Province, 350005, China

**Keywords:** intervertebral disc degeneration, LINC00689, MiR-3127-5p, ATG7, autophagy

## Abstract

This study aimed to explore the effects of long non-coding RNAs LINC00689 (LINC00689) in human nucleus pulposus cells (NPCs). NPCs were isolated and their morphology was observed. The proliferation and apoptosis of NPCs, and the levels of LINC00689, miR-3127-5p, Bax, Bcl-2, Cleaved caspase-3, ATG5, ATG7, p62, and LC3Ⅱ/LC3I were detected. Interrelations of LINC00689, miR-3127-5p, and ATG7 were analyzed. LINC00689 was down-regulated yet miR-3127-5p was up-regulated in NPCs. LINC00689 could competitively bind with miR-3127-5p, and ATG7 was targeted by miR-3127-5p in NPCs. Overexpressed LINC00689 promoted proliferation yet inhibited apoptosis of NPCs, whereas LINC00689 silencing did the opposite. Overexpressed LINC00689 raised ATG7 level and LC3Ⅱ/LC3I value yet reduced that of p62 level, but the depletion of LINC00689 did the contrary. ATG7 silencing abolished the effects of overexpressed LINC00689 in NPCs, and likewise, up-regulation of miR-3127-5p overturned the effects of overexpressed LINC00689 in NPCs. Collectively, the up-regulation of LINC00689 inhibits the apoptosis of NPCs via miR-3127-5p/ATG7 axis-mediated autophagy.

## 1 Introduction

The intervertebral disc (IVD) is an important part of the spinal column and plays a key role in the spinal movement and the intervertebral juncture in general [1]. As a typical common disease in clinical practice, IVD degeneration (IDD) is the pathological basis of spinal degenerative diseases, which can cause a series of clinical syndromes such as disc herniation, low back pain, and cervical spondylosis [2–4]. Relevant investigation has shown that various diseases caused by IDD tremendously decrease the life quality of patients [5]. Currently, many treatment modalities for IDD have been developed, including tissue engineering, stem cell injections, and therapeutic protein administrations [6]. In addition to the discovery highlighting IDD as the result of a combination of factors, recent studies have further demonstrated that genetic factors play an important role in the occurrence of IDD [7]. Therefore, gene therapy for IDD has received increasing attention and research in recent years [8].

Increasing findings have shown that abnormal expression of genetic factors occurs in the development and progression of IDD [9]. Long non-coding RNAs (lncRNAs) are a class of non-coding RNAs with transcripts more than 200 nucleotides in length [10]. LncRNAs do not code for proteins, resulting in their lack of attention by scholars in the initial period of time; however, in recent years, a large number of studies have proposed the involvement of lncRNAs in the progression of various diseases [11,12]. Similarly, lncRNAs participated in the progression of osteoarthritis and IDD, such as lncRNA HOTAIR, lncRNA TUG1, and lncRNA MAGI2-AS3 [13–15]. Moreover, a previous study has reported that lncRNA LINC00689 expression was down-regulated in IDD [9]. Nevertheless, its effect in IDD awaits to be further elucidated.

In accordance with the results of the existing study, the involvement of lncRNA–miRNA–mRNA network in IDD has been indicated [9]. A previous study has profiled that lncRNA prostate androgen-regulated transcript 1 (lncRNA PART1) expression was increased in lipopolysaccharide-treated human nucleus pulposus cells (NPCs) and that the up-regulation of lncRNA PART1 promoted the progression of IDD via targeting miR-190a-3p [16]. Interestingly, the up-regulated expression of miR-3127-5p in IDD samples has been evidenced, suggesting that miR-3127-5p may also be involved in the development of IDD [9]. What additionally caught our attention is the discovery that Metformin increased paclitaxel sensitivity of ovarian cancer cells via miR-3127-5p-mediated autophagy [17]. Meanwhile, it has been documented in several studies that the senescence of NPCs plays a vital role in the pathogenesis and development of IDD [18,19]. Furthermore, a number of researchers have reported that activating the autophagy of NPCs reduced cellular senescence and apoptosis [20,21]. Therefore, we speculated that the down-regulation of LINC00689 could promote the apoptosis of NPCs via miR-3127-5p-mediated autophagy.

In this work, we evaluated the expressions of LINC00689 and miR-3127-5p in NP tissues and cells. Moreover, we investigated the interaction between LINC00689 and miR-3127-5p in the autophagy of NPCs, with the hope to provide new insights into the gene therapy for IDD.

## 2 Materials and methods

### 2.1 Ethics statement and tissue samples collection

This study was approved by the Ethics Committee of First Affiliated Hospital of Fujian Medical University (ZL2020080215). Ten degenerative IVD NP tissues (IDD group) were obtained from ten patients (including five females and five males, with a mean age of 54 ± 10 years old) with lumbar disc herniation (LDH). All patients were diagnosed with LDH and underwent lumbar spine surgery at the Orthopedic Department of our hospital between September 2020 and February 2021. Meanwhile, ten normal IVD NP tissues were obtained from ten patients (including five females and five males, with a mean age of 52 ± 10 years old) with lumbar vertebrae fractures (LVF). The informed consent was obtained from all patients before tissue samples were collected.

### 2.2 Extraction, culture, and observation of NPCs

The NPCs were isolated using the method as previously described [4]. First, the collected NP tissues were cut with a size of 1 mm^3^ and incubated with trypsin (0.25%, PB180228, Procell, Wuhan, China) for 30 min, followed by centrifugation at 1,000*g* (E2658, Beyotime, Shanghai, China) for 10 min and incubation with collagenase type II (40508ES60, Qcbio Science & Technologies Co., Ltd, Shanghai, China) at 37°C for 4 h. Later, the treated NP tissues were filtered with a 200-mesh filter (S4203, Aladdin, Shanghai, China), maintained in Dulbecco’s Modified Eagle Medium/Nutrient Mixture F medium (with the inclusion of 1% penicillin–streptomycin mixture solution, PM150312A, Procell, Wuhan, China) supplemented with 20% of fetal bovine serum (164210, Procell, Wuhan, China) and cultured in an incubator (BC-J80, BoXun, Shanghai, China) at 37°C with 5% CO_2_. The cell medium was replaced 2–3 times a week.

When the NPCs grew attached, the morphology of primary NPCs was observed (magnification 200×) under an inverted microscope (Ts2-FL, Nikon, Tokyo, Japan) as appropriate. NPCs with passage ≤3 (*P* ≤ 3) were used for the subsequent experiments in the present study [22–24].

### 2.3 Bioinformatic analysis

The upstream miRNAs of autophagy related 7 (ATG7) were analyzed by the following websites, including: Starbase (http://starbase.sysu.edu.cn/index.php), TargetScan (http://www.targetscan.org/vert_72/), and LncBase Predicted v.2 (http://carolina.imis.athena-innovation.gr/diana_tools/web/). Following the analysis of the data, we obtained five potential upstream miRNAs of ATG7 (miR-3127-5p, miR-769-5p, miR-3179, miR-129-5p, and miR-766-5p). Moreover, the interrelations of LINC00689, miR-3127-5p, and ATG7 were predicted by Targetscan and Starbase.

### 2.4 Cell transfection

The small interfering RNA specifically targeting LINC00689 (siLINC00689) and its negative control (siNC), the overexpression plasmid of LINC00689 and its negative control (NC, pcDNA3.1 vector), as well as the small interfering RNAs targeting ATG7 (siATG7) were synthesized and purchased from GenePharma (Shanghai, China). Besides, miR-3127-5p mimic (abbreviated as M in the figures, miR10014990-1-5) and mimic control (abbreviated as MC in the figures, miR1N0000002-1-5) were ordered from RiboBio (Guangzhou, China).

For cell transfection, NPCs were grown in 6-well plates (5 × 10^5^ cells/well). Then, cells at a confluence of 80% were transfected with siLINC00689 (100 pmol), siNC (100 pmol), LINC00689 (5 μg), NC (5 μg), siATG7 (100 pmol), miR-3127-5p M (100 nM), or MC (100 nM) at room temperature for 48 h, which was performed with the help of lipofectamine 3000 reagent (L3000015, Thermo Fisher Scientific, Waltham, MA, USA).

### 2.5 Dual-luciferase reporter assay

The binding sites in between ATG7 and miR-3127-5p and between LINC00689 and miR-3127-5p were confirmed by dual-luciferase reporter assay kit (RG027, Beyotime, Shanghai, China). The wild-type sequence of ATG7 (ATG7-WT: 5′-GAUCCUUUCCCCUUGGCCCUGAG-3′), mutant sequence of ATG7 (ATG7-MUT: 5′-GAUCCUUUCCCCUUGGCCCCAAG-3′), wild type sequence of LINC00689 (LINC00689-WT: 5′-CGACUGGAGGGUCUUGCCCUGAG-3′), and mutant sequence of LINC00689 (LINC00689-MUT: 5′-CGACUGGAGGGUCUUGAACUCAG-3′) were structured and sub-cloned into pGL3 luciferase reporter vectors by GenePharma Company (Shanghai, China).

To verify the relationship between ATG7 and miR-3127-5p, HEK293T cells (CL-0005, Procell, Wuhan, China) were co-transfected with 0.25 μg ATG7-WT/ATG7-MUT plasmids and 50 nM miR-3127-5p mimic/mimic control. Likewise, to verify the relationship between LINC00689 and miR-3127-5p, HEK293T cells were co-transfected with 0.25 μg LINC00689-WT/LINC00689-MUT plasmids and 50 nM miR-3127-5p mimic/mimic control. Following the culture of HEK293T cells for 48 h, the relative luciferase activity was evaluated using the dual-luciferase reporter assay kit and the microplate reader (GM3000, Promega, Madison, WI, USA).

### 2.6 Cell proliferation assay

The proliferation of NPCs was measured by the EdU Cell Proliferation Kit (C0071S, Beyotime, Shanghai, China). Prior to this assay, the Click Additive Solution, EdU reagent, and Hoechst 33342 reagent were prepared with the help of EdU Cell Proliferation Kit. NPCs were maintained in 6-well plates (5 × 10^5^ cells/well) and transfected as instructed. Cells were then incubated with EdU reagent at 37°C for 2 h, after which these cells were fixed with 4% paraformaldehyde (P0099, Beyotime, Shanghai, China) and washed with wash buffer as appropriate. Afterwards, cells were incubated with immunol staining wash buffer (P0106, Beyotime, Shanghai, China) at room temperature for 15 min, and then treated with Click Additive Solution for 30 min in the dark, followed by the staining with Hoechst 33342 reagent at room temperature for 10 min in the dark. Finally, cells were washed with wash buffer and observed (magnification 200×) under a fluorescence microscope (MVX10, OLYMPUS, Tokyo, Japan).

### 2.7 Cell apoptosis assay

In this assay, the apoptosis of NPCs was measured using the Annexin V-FITC/Propidium Iodide Apoptosis Detection Kit (C1062M, Beyotime, Shanghai, China). Specifically, the treated NPCs were washed with PBS (C0221A, Beyotime, Shanghai, China), digested with trypsin solution, and resuspended with PBS. An appropriate amount (5 × 10^4^) of NPCs was resuspended with 195 μL AnnexinV-FITC conjugated solution, and then the cells were treated with 5 μL of Annexin V-FITC and 10 μL of propidium iodide at room temperature for 15 min. Finally, the flow cytometer (CytoFLEX, Beckman Coulter, Inc., Kraemer Boulevard Brea, CA, USA) was used to assess the apoptosis of NPCs, and the results were analyzed with the help of Kaluza C software (v. 1.1.2, Beckman Coulter, Indianapolis, IN, USA).

### 2.8 Quantitative RT-PCR (qRT-PCR)

In this work, qRT-PCR was performed on the qRT-PCR system (ABI7700, Applied Biosystems, Carlsbad, CA, USA). The tissue samples and transfected NPCs were harvested prior to the analysis of qRT-PCR. TransZol Up Plus RNA Kit (ER501-01) was purchased from TransGen Biotech (Beijing, China) and employed to extract the total RNA from the collected tissues and cells. The concentration of isolated RNA samples was evaluated using a spectrophotometer (Cary 60 UV-Vis, Agilent, Santa Clara, CA, USA). Next, using RNA as template, the cDNA was synthesized with the help of First-Strand Synthesis System (18091050, Thermo Fisher Scientific, Waltham, MA, USA), and the reaction mix solution for qRT-PCR was prepared by the Top Green qPCR SuperMix kit (AQ131-01, TransGen Biotech, Beijing, China). After the supplementation of cDNA synthesized above and the corresponding primers (Table 1), the qRT-PCR reaction mix solution was detected by the qRT-PCR system. The results in our study were analyzed with 2^−ΔΔct^ method [25], and GAPDH or U6 was used as the endogenous control.

**Table 1:** All primers in qRT-PCR experiments in this study

ID	Forward sequence (5′−3′)	Reverse sequence (5′−3′)
**LINC00689**	AGTTGGTACAGGGAGGGGTT	GTCCCTCTTGGTGGAGTTGG
**miR-3127-5p**	CGGGCTTGTGGAATGGTAAGC	CTGTCAGCTTCCCATTCC
**U6**	CTCGCTTCGGCAGCACA	AACGCTTCACGAATTTGCGT
**GAPDH**	GGAGCGAGATCCCTCCAAAAT	GGCTGTTGTCATACTTCTCATGG

### 2.9 Western blot

The treated NPCs were harvested first, and the total protein was subsequently extracted from the treated cells (5 × 10^6^) with the help of total protein extraction kit (C1396, Jining Shiye, Shanghai, China), after which the concentration of protein samples was calculated using the BCA protein assay kit (C1397, Jining Shiye, Shanghai, China). Later, SDS-PAGE gel (BB-3702, BestBio, Nanjing, China) was prepared and 20 μL of protein samples was electrophoresed on the prepared SDS-PAGE gel. Subsequently, the separated proteins were transferred onto the PVDF membrane (1620177) ordered from Bio-Rad (Hercules, CA, USA). Later, the PVDF membrane was blocked with Blocker™ BLOTTO TBS Buffer (37530, Thermo Fisher Scientific, Waltham, MA, USA) at room temperature for 1 h, and then washed with Western Wash Buffer (P0023C3, Beyotime, Shanghai, China). Next, the PVDF membrane was incubated with diluted solution of primary antibodies at 4°C overnight, and then incubated with secondary antibodies at room temperature for 1 h. Lastly, PVDF membrane was visualized by ECL solution (1705062, Bio-Rad, Hercules, CA, USA), and the results were analyzed using the Western blot imaging system (ChemiDoc XRS+, Bio-Rad, Hercules, CA, USA). The information of all antibodies used in this research is listed in Table 2.

**Table 2:** All antibodies information and sources in Western blot in this study

ID	Catalog number	Company (country)	Molecular weight (kDa)	Dilution ratio
Bax	ab182733	Abcam (Cambridge, UK)	21	1/2,000
Bcl-2	ab182858	Abcam (Cambridge, UK)	26	1/2,000
Cleaved caspase-3	ab32042	Abcam (Cambridge, UK)	17	1/500
ATG5	ab108327	Abcam (Cambridge, UK)	32	1/1,000
ATG7	ab52472	Abcam (Cambridge, UK)	70	1/10,000
P62	ab109012	Abcam (Cambridge, UK)	62	1/10,000
LC3B	ab192890	Abcam (Cambridge, UK)	14, 16	1/2,000
GAPDH	ab181602	Abcam (Cambridge, UK)	36	1/10,000
Rabbit IgG	ab205718	Abcam (Cambridge, UK)		1/5,000

### 2.10 Statistical analysis

In this study, all measured data were described as mean ± standard deviation (SD). The data of the LVF group and IDD group were compared by independent sample *t*-test. One-way analysis of variance (ANOVA) was used for the comparison among multiple groups, and Pearson’s correlation analysis was applied to evaluate the correlation between the expression levels of LINC00689 and miR-3127-5p. All statistical analyses were implemented using GraphPad 8.0 software. The data with *P* < 0.05 were considered as statistically significant.

## 3 Results

### 3.1 LINC00689 was down-regulated in IDD tissue, and overexpressed LINC00689 promoted the proliferation yet inhibited the apoptosis of NPCs, whereas LINC00689 silencing did the opposite

**Figure 1:**
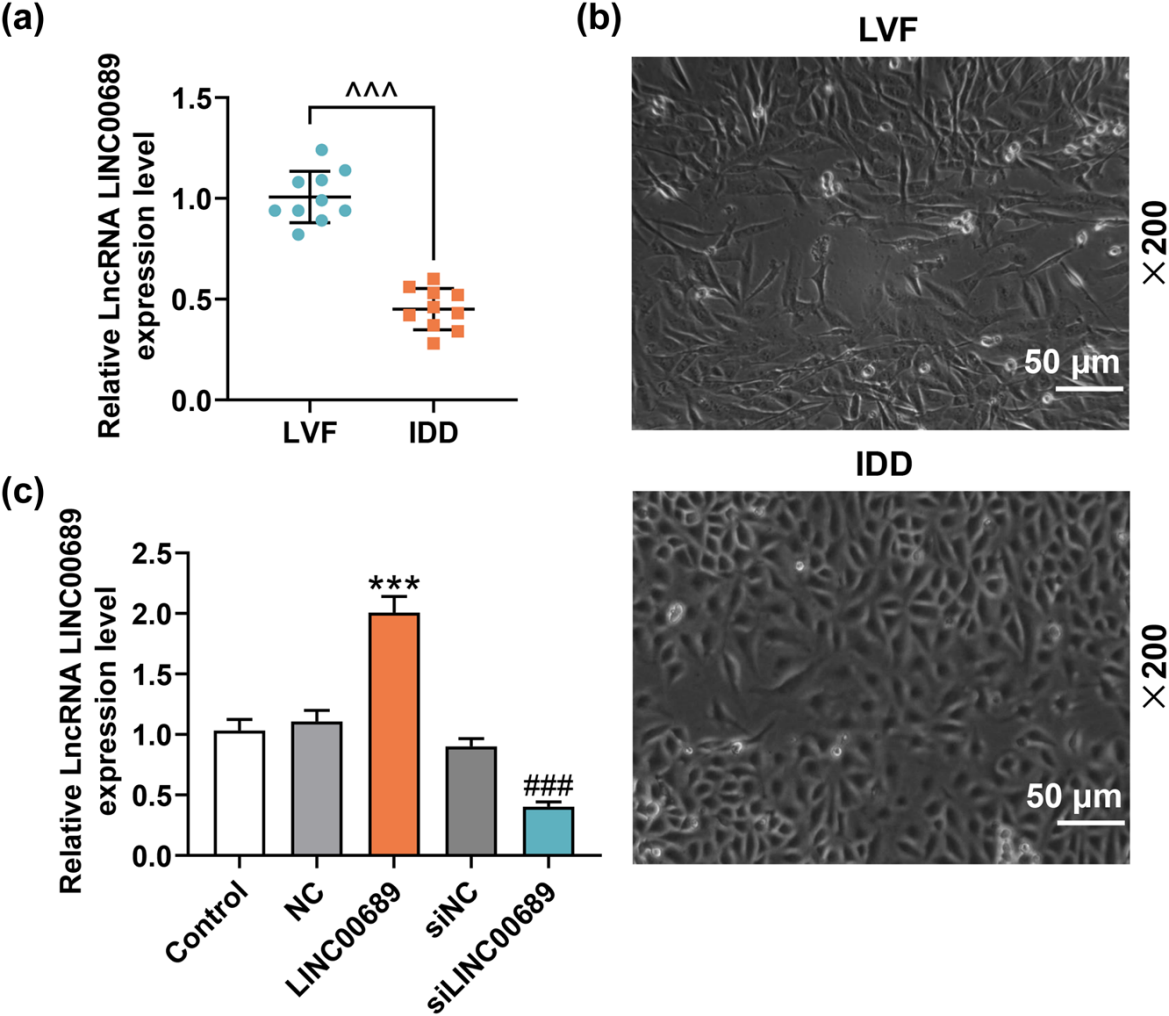
The level of LINC00689 was down-regulated in IDD tissue. (a) The level of LINC00689 in NP tissue samples was measured by qRT-PCR, and the expression of LINC00689 was lower in IDD group than that in LVF group. (b) The NPCs were extracted from collected NP tissues, and then the cell morphology of NPCs was observed under an inverted microscope (under 200× magnification, scale bar = 50 μm). (c) The expression of LINC00689 in transfected NPCs was measured by qRT-PCR. ^^^^^ *P* < 0.001 vs LVF; ^***^ *P* < 0.001 vs NC; ^###^ *P* < 0.001 vs siNC (LINC00689: long non-coding RNA LINC00689, IDD: intervertebral disc degeneration, LVF: lumbar vertebrae fractures, NPCs: human nucleus pulposus cells, NP: nucleus pulposus, siLINC00689: small interfering RNA specifically targeting LINC00689, NC: negative control, qRT-PCR: quantitative RT-PCR).

The NP tissues were collected at first, and the level of LINC00689 in the tissue samples was then measured by qRT-PCR. The results suggested that the level of LINC00689 was down-regulated in IDD group as compared with that in LVF group (Figure 1a, *P* < 0.001). Next, the NPCs were extracted from the tissue samples and then the morphology of NPCs was observed as appropriate. It was seen that the morphology of NPCs in LVF group was mostly polygonal and fusiform, whereas that of NPCs in IDD group was mostly irregular and fusiform (Figure 1b). As such, these NPCs which had been isolated from NP tissues in IDD group were collected for the following study.

**Figure 2:**
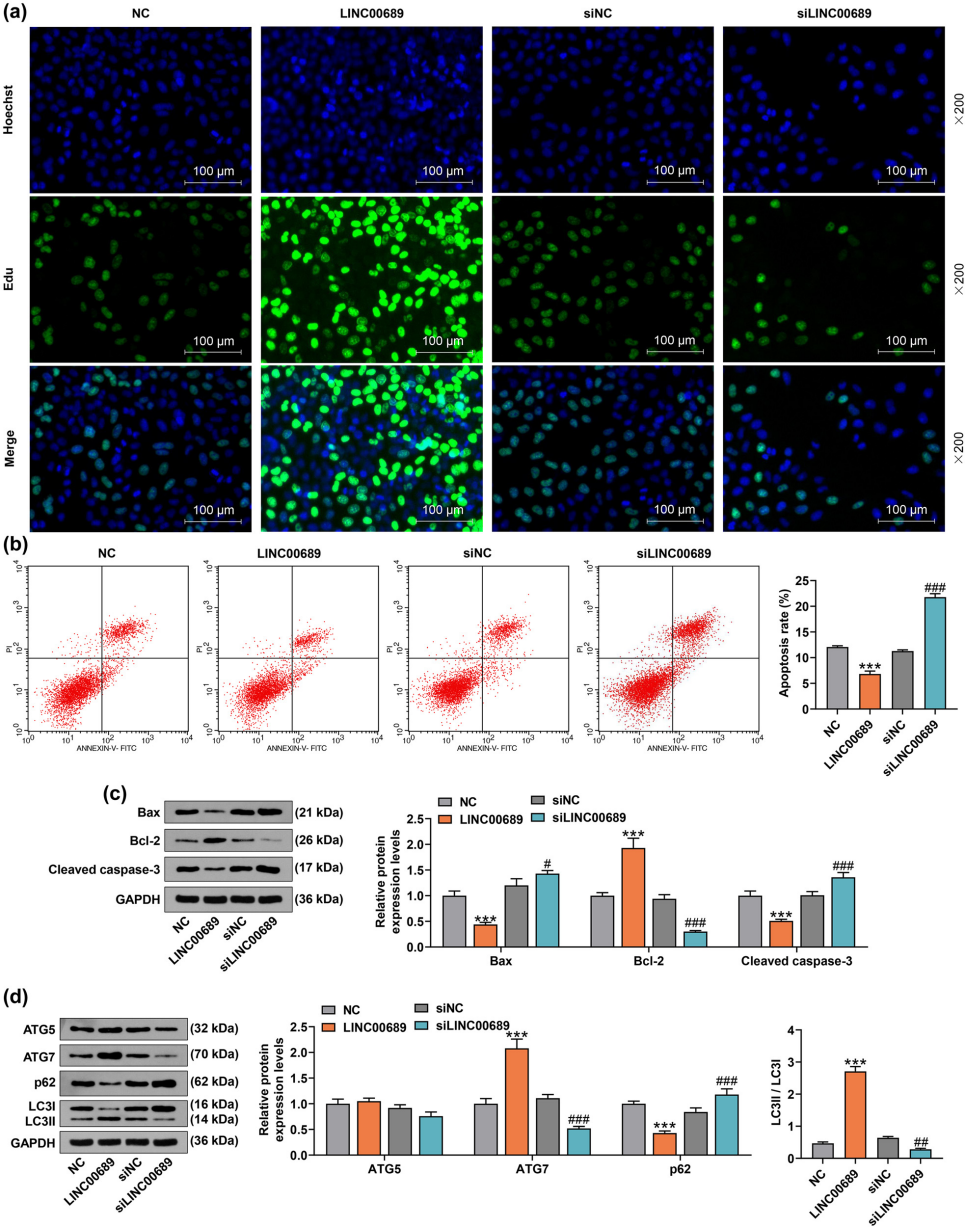
Overexpressed LINC00689 promoted the proliferation and autophagy yet inhibited the apoptosis of NPCs, whereas siLINC00689 did the opposite. (a) The proliferation of NPCs was measured by EdU staining after NPCs were transfected with LINC00689 overexpression plasmid or siLINC00689 (under 200× magnification, scale bar = 100 μm). (b) The apoptosis of NPCs was detected by flow cytometry. (c) The expressions of apoptosis-related factor (Bax, Bcl-2, and Cleaved caspase-3) in transfected NPCs were examined using the Western blot. (d) The levels of autophagy-related proteins (ATG5, ATG7, p62, and LC3Ⅱ/LC3I) in transfected NPCs were analyzed by the Western blot. ^***^ *P* < 0.001 vs NC; ^#^ *P* < 0.05, ^##^ *P* < 0.01, ^###^ *P* < 0.001 vs siNC (LINC00689: long non-coding RNAs LINC00689, NPCs: human nucleus pulposus cells, siLINC00689: small interfering RNA specifically targeting LINC00689, NC: negative control, ATG7: autophagy related 7).

NPCs were transfected with LINC00689 overexpression plasmids or siLINC00689 or the corresponding NC as needed. The results of qRT-PCR showed that LINC00689 expression was up-regulated by the overexpression plasmids of LINC00689 yet down-regulated by siLINC00689 (Figure 1c, *P* < 0.001). Subsequently, we found that the number of EdU-positive cells was elevated by the overexpression plasmids of LINC00689 and decreased by siLINC00689, suggesting that the proliferation of NPCs was promoted by the overexpression of LINC00689 yet inhibited by the silence of LINC00689 (Figure 2a). Furthermore, overexpressed LINC00689 remarkably reduced the apoptosis of NPCs, while LINC00689 silencing evidently accelerated the apoptosis of NPCs (Figure 2b, *P* < 0.001). In addition, we examined the expressions of apoptosis-related factors (Bax, Bcl-2, and Cleaved caspase-3) in the transfected NPCs. It was observed in these results that overexpressed LINC00689 inhibited the levels of Bax and Cleaved caspase-3, while promoting the level of Bcl-2 (Figure 2c, *P* < 0.001). On the contrary, LINC00689 silencing increased the levels of Bax and Cleaved caspase-3, but decreased the level of Bcl-2 in NPCs (Figure 2c, *P* < 0.05). These data demonstrated that overexpressed LINC00689 promoted the proliferation yet inhibited the apoptosis of NPCs, whereas LINC00689 silencing exerted the opposite effects. Therefore, we hypothesized that LINC00689, with an aberrant expression, was involved in the progression of IDD.

### 3.2 Overexpressed LINC00689 inhibited the apoptosis of NPCs via activating ATG7-dependent canonical autophagy in NPCs, whereas LINC00689 silencing exerted the opposite effect

**Figure 3:**
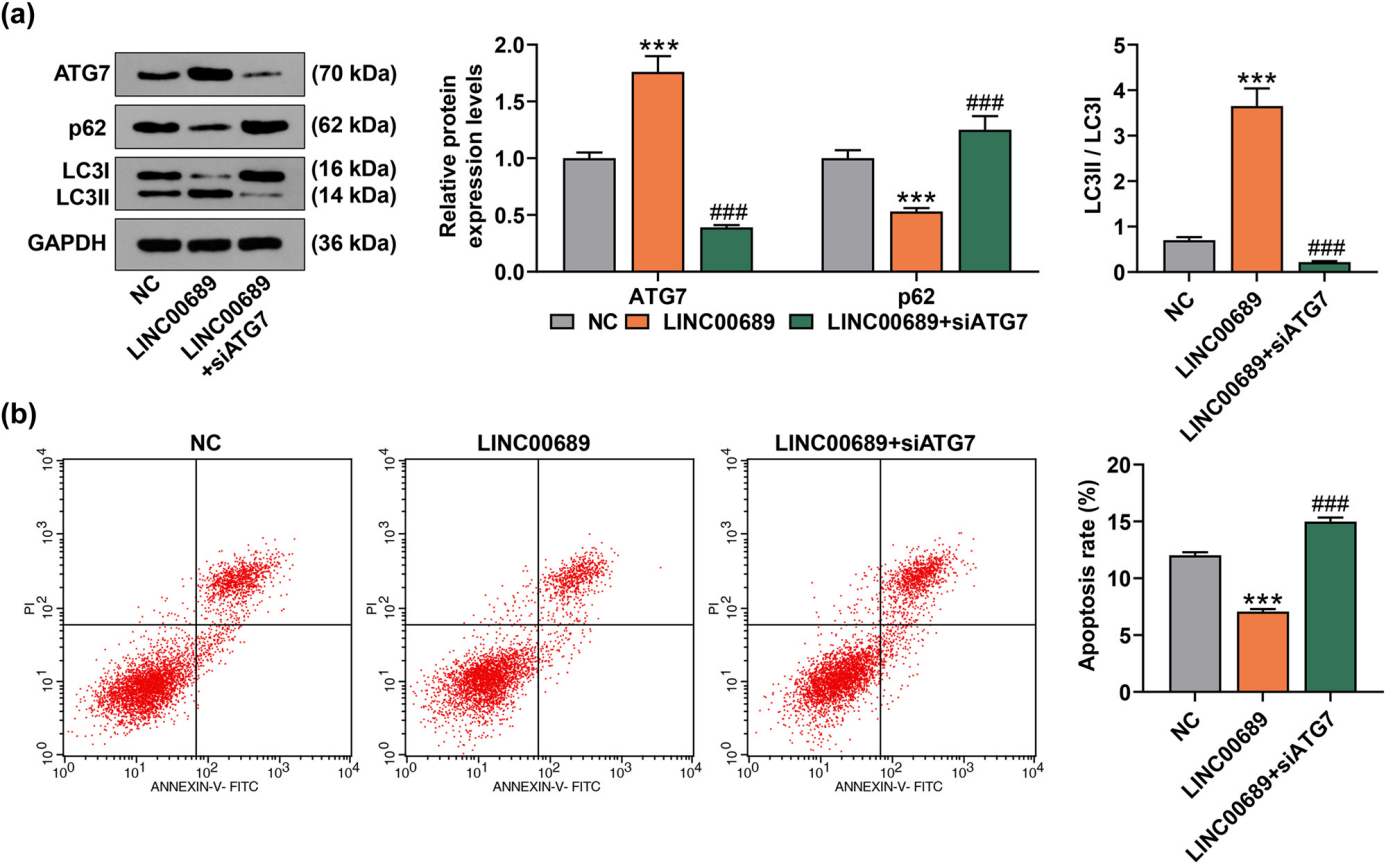
SiATG7 abolished the effect of overexpressed LINC00689 on promoting the autophagy and on inhibiting the apoptosis of NPCs. (a) The levels of autophagy-related proteins (ATG7, p62, and LC3Ⅱ/LC3I) were quantified by the Western blot. (b) Following the transfection of NPCs with LINC00689 overexpression plasmid and siATG7, the apoptosis of NPCs was detected by using flow cytometry. ^***^ *P* < 0.001 vs NC; ^###^ *P* < 0.001 vs LINC00689 (NPCs: human nucleus pulposus cells, ATG7: autophagy related 7).

Activation of autophagy in NPCs has been found to decrease cellular senescence and apoptosis [26]. In light of this, we measured the levels of autophagy-related proteins (ATG5, ATG7, p62, and LC3II/LC3I) in transfected NPCs. As shown in Figure 2d, the overexpression of LINC00689 raised the ATG7 level and LC3II/LC3I value, while reducing the p62 level (*P* < 0.001). However, the knockdown of LINC00689 markedly weakened ATG7 level and LC3II/LC3I value, but elevated the p62 level in NPCs (Figure 2d, *P* < 0.01). Furthermore, ATG7 silencing abolished the effects of overexpressed LINC00689 on promoting ATG7 level and LC3II/LC3I value and on inhibiting p62 expression (Figure 3a, *P* < 0.001). Subsequently, the apoptosis of transfected NPCs was analyzed, the results of which showed that the overexpression of LINC00689 notably repressed the apoptosis of NPCs (*P* < 0.001). Conversely, the knockdown of ATG7 decreased the suppressive effect of overexpressed LINC00689 on the apoptosis of NPCs (Figure 3b, *P* < 0.001). These data herein suggested that overexpressed LINC00689 inhibited the apoptosis of NPCs via activating ATG7-dependent canonical autophagy in NPCs, whereas LINC00689 silencing exerted the opposite effect.

### 3.3 LINC00689 could competitively bind with miR-3127-5p, and ATG7 was targeted by miR-3127-5p in NPCs

**Figure 4:**
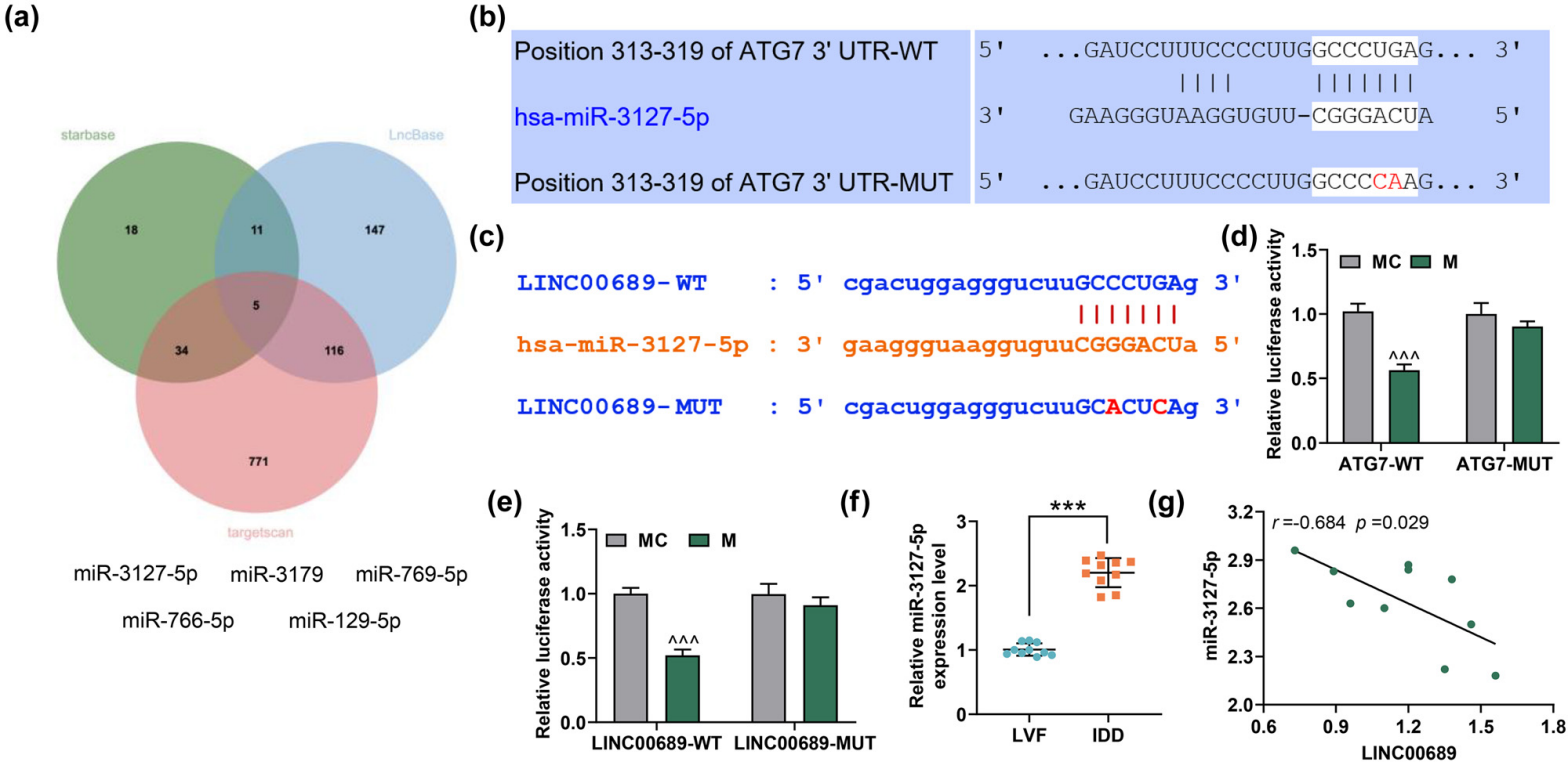
LINC00689 could competitively bind with miR-3127-5p, and ATG7 was targeted by miR-3127-5p in NPCs. (a) The upstream miRNAs of ATG7 were predicted by Starbase, TargetScan, and LncBase Predicted v.2 websites. By analyzing the data, five potential upstream miRNAs of ATG7 (miR-3127-5p, miR-769-5p, miR-3179, miR-129-5p, and miR-766-5p) were obtained. (b) TargetScan was used to predict the binding site of miR-3127-5p and ATG7. (c) The binding site of LINC00689 and miR-3127-5p was predicted via Starbase. (d and e) The interrelations of LINC00689, miR-3127-5p, and ATG7 were analyzed using dual-luciferase reporter assay. (f) The level of miR-3127-5p in NP tissue samples was measured by qRT-PCR. (g) The correlation between miR-3127-5p and LINC00689 levels was analyzed. ^^^^^ *P* < 0.001 vs MC; ^***^ *P* < 0.001 vs LVF (NPCs: human nucleus pulposus cells, qRT-PCR: quantitative RT-PCR, M: miR-3127-5p mimic, MC: mimic control, LVF: lumbar vertebrae fractures, IDD: intervertebral disc degeneration, ATG7: autophagy related 7, NP: nucleus pulposus).

As shown in Figure 4a, five potential upstream miRNAs that could target ATG7 were obtained, namely, miR-3127-5p, miR-769-5p, miR-3179, miR-129-5p, and miR-766-5p. A previous study has reported the high expression of miR-3127-5p in lumbar IDD [9]. Here, the complementary binding sites in between LINC00689/ATG7 and miR-3127-5p are shown in Figure 4b and c. Moreover, we found that the luciferase activity was decreased when NPCs were co-transfected with ATG7-WT or LINC00689-WT and miR-3127-5p mimic (Figure 4d and e, *P* < 0.001). Besides, there was no difference in the luciferase activity when NPCs were co-transfected with ATG7-MUT or LINC00689-MUT and miR-3127-5p mimic or mimic control. These results thus signified that ATG7 was indeed targeted by miR-3127-5p, and miR-3127-5p was further targeted by LINC00689 in NPCs. Additionally, miR-3127-5p expression was proved to be up-regulated in NP tissues of the IDD group when compared with that in LVF group (Figure 4f, *P* < 0.001). It was clearly mirrored in correlation analysis that LINC00689 expression was negatively correlated with miR-3127-5p expression in IDD tissues (Figure 4g, *r* = −0.684, *P* = 0.029). These results confirmed that LINC00689 could competitively bind with miR-3127-5p, the miRNA which could target ATG7 in IDD.

### 3.4 Up-regulation of miR-3127-5p reversed the effects of overexpressed LINC00689 on promoting the proliferation and autophagy and on inhibiting the apoptosis of NPCs

**Figure 5:**
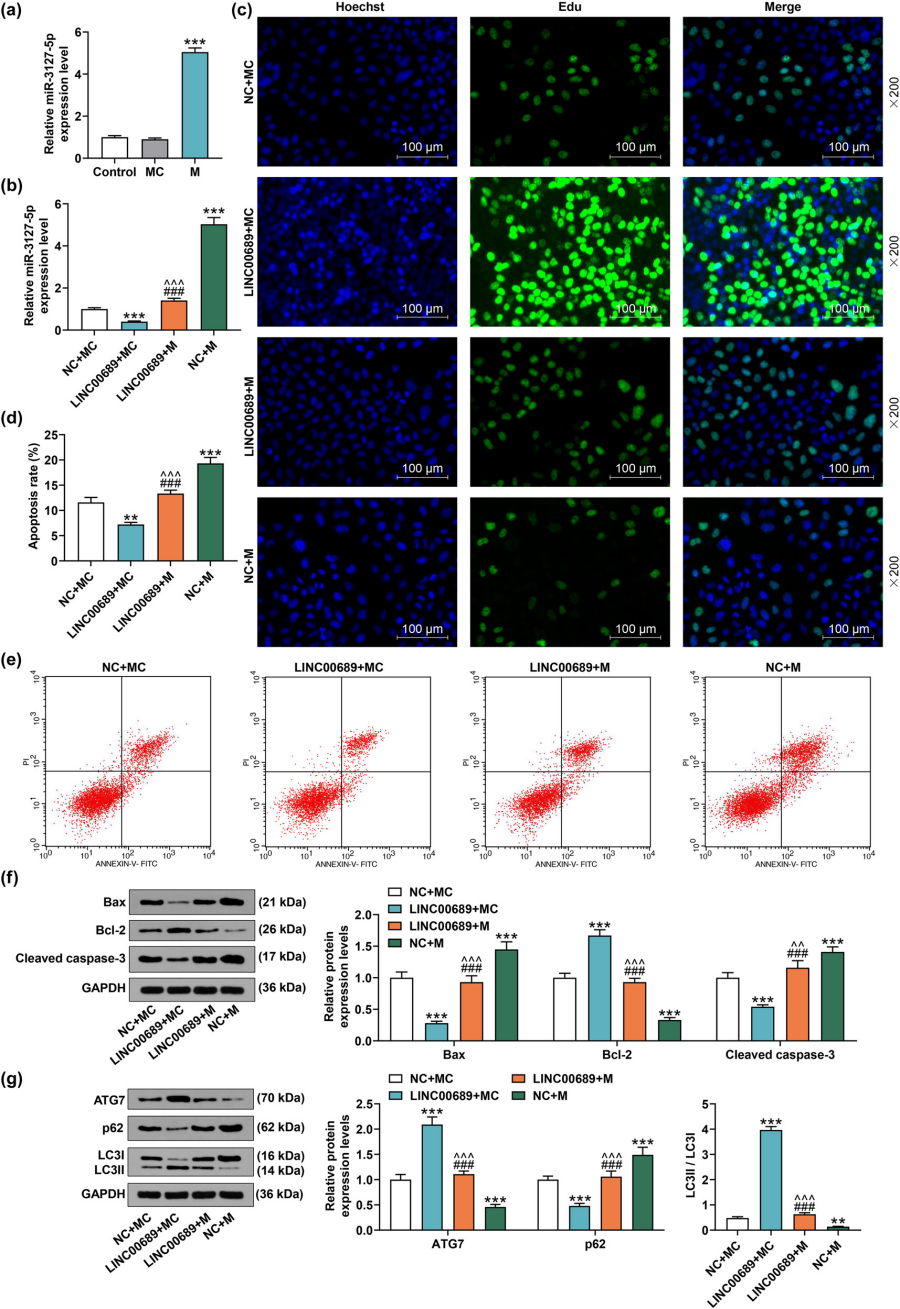
Up-regulation of miR-3127-5p reversed the effect of overexpressed LINC00689 on promoting the proliferation and autophagy, and on inhibiting the apoptosis of NPCs. (a and b) The expression of miR-3127-5p in transfected NPCs was analyzed using the qRT-PCR. (c) The proliferation of NPCs was measured by EdU staining (under 200× magnification, scale bar = 100 μm). (d and e) The apoptosis of NPCs was detected by flow cytometer. (f) The expressions of apoptosis-related factors (Bax, Bcl-2, and Cleaved caspase-3) in transfected NPCs were examined using the Western blot. (g) The levels of autophagy-related proteins (ATG7, p62, and LC3Ⅱ/LC3I) in transfected NPCs were analyzed by the Western blot. ^**^ *P* < 0.01, ^***^ *P* < 0.001 vs MC (Figure 5a) or NC + MC (Figure 5b–g); ^^^^ *P* < 0.01, ^^^^^ *P* < 0.001 vs LINC00689 + MC; ^###^ *P* < 0.001 vs NC + M. (LINC00689: long non-coding RNAs LINC00689, NPCs: human nucleus pulposus cells, M: miR-3127-5p mimic, MC: mimic control, NC: negative control, ATG7: autophagy related 7).

The results of qRT-PCR manifested that miR-3127-5p mimic notably enhanced the level of miR-3127-5p in NPCs (Figure 5a, *P* < 0.001). As depicted in Figure 5b, overexpressed LINC00689 inhibited the expression of miR-3127-5p, while miR-3127-5p mimic enhanced miR-3127-5p level and reversed the inhibitory effect of LINC00689 overexpression on miR-3127-5p expression in NPCs (Figure 5b, *P* < 0.001). Meanwhile, LINC00689 overexpression obviously facilitated the proliferation of NPCs, while overexpressed miR-3127-5p inhibited the proliferation of NPCs (Figure 5c). Additionally, the up-regulation of miR-3127-5p reversed the effect of LINC00689 overexpression on promoting the proliferation of NPCs (Figure 5c). Moreover, the apoptosis of NPCs was reduced following the overexpression of LINC00689 yet raised by the up-regulation of miR-3127-5p, and more importantly, the up-regulation of miR-3127-5p reversed the effect of overexpressed LINC00689 on inhibiting the apoptosis of NPCs (Figure 5d and e, *P* < 0.001).

The results of Western blot displayed that overexpressed LINC00689 suppressed the levels of Bax and Cleaved caspase-3 and promoted that of Bcl-2 in NPCs, while the overexpression of miR-3127-5p up-regulated the levels of Bax and Cleaved caspase-3 yet down-regulated the levels of Bcl-2 in NPCs (Figure 5f, *P* < 0.001). Also, the up-regulation of miR-3127-5p counteracted the effects of overexpressed LINC00689 on inhibiting the levels of Bax and Cleaved caspase-3 yet promoting the Bcl-2 level in NPCs (Figure 5f, *P* < 0.01). In addition, the up-regulation of LINC00689 raised the ATG7 level and LC3Ⅱ/LC3I value, while reducing p62 level (Figure 5g, *P* < 0.001). However, the overexpression of miR-3127-5p not only led to the inhibited ATG7 level and LC3Ⅱ/LC3I ratio and the promoted p62 level, but also reversed the effects of LINC00689 overexpression on increasing the ATG7 level and LC3Ⅱ/LC3I ratio yet decreasing the p62 expression in NPCs (Figure 5g, *P* < 0.001). These results suggested that up-regulation of miR-3127-5p has the ability to reverse the effects of LINC00689 overexpression on promoting the proliferation and autophagy and on inhibiting the apoptosis of NPCs.

## 4 Discussion

IDD is one of the main causes of back pain [27]. The IVD is comprised of the inner NP, which is encircled by the cartilaginous endplates and annulus fibrosis lying between the adjacent vertebral bodies and IVD [28]. It has already been suggested that the functional changes of NPCs are considered to be the initiating factors of IDD [28]. Additionally, current research has already shown that the molecular biological process of IDD is abnormally complex and that numerous cytokines and proteins, such as inflammatory factors, growth factors, and matrix-degrading enzymes, are abnormally expressed at the molecular level [27,29,30].

A great deal of researchers have reported that lncRNAs are involved in numerous processes, with the regulatory effects on gene expression [31]. In addition, dysregulated expression of lncRNAs is closely linked to many human diseases, such as neurological diseases, cancer, osteoarthritis, and IDD [32]. Similarly, aberrantly expressed lncRNAs are involved in the initiation and development of IDD by regulating the abnormal phenotypes of NPCs, the proliferation and apoptosis of cells, for instance [33]. In this study, some significant changes concerning the morphology of NPCs have been evidenced in IDD patients. It has been reported that lncRNA ANPODRT expression was reduced in degenerative NP tissues and that lncRNA ANPODRT inhibited the apoptosis of NPCs via activating Nrf2 signaling [34]. Chen et al. have demonstrated that LINC00324 level was increased in IDD patients, and LINC00324 may accelerate the IDD progression via up-regulating the expression of Fas ligand [35]. Also, recent evidence has additionally suggested that lncRNA LINC00689 expression was down-regulated in IDD, despite its vague effect in IDD, which awaited to be further elucidated [9]. In this research, we also found that LINC00689 expression was down-regulated in IDD tissue. Apart from this, for the first time, we found that overexpressed LINC00689 promoted the proliferation yet inhibited the apoptosis of NPCs, whereas LINC00689 silencing did the opposite. These results suggested that LINC00689 indeed regulated the biological behaviors of NPCs.

To further validate the experimental results above, we examined the expressions of apoptosis-related factors (Bax, Bcl-2, and Cleaved caspase-3) in treated NPCs as needed [36]. Bcl-2 family plays a vital role in the intrinsic apoptosis of cells. Bax and Bcl-2 belong to the Bcl-2-related family, in which Bcl-2 is an apoptosis inhibitor, while Bax is an apoptosis promoter [37,38]. Moreover, the cell apoptosis has been proposed to be orchestrated by caspases family, among which caspase-3 is responsible for the majority of proteolysis during apoptosis, making Cleaved caspase-3 level thereby considered as a marker for evaluating the apoptosis of cells [39]. In this study, we discovered that overexpressed LINC00689 inhibited the levels of Bax and Cleaved caspase-3, while promoting Bcl-2 level. On the contrary, the silence of LINC00689 increased the levels of Bax and Cleaved caspase-3 yet decreased the level of Bcl-2 in NPCs. In the previous studies it has been suggested that activating the autophagy of NPCs reduced cell senescence and apoptosis [20,21]. ATG5 and ATG7 are thought to be essential for the induction of autophagy [40]. A recent evidence has profiled that miR-210 promoted extracellular matrix degradation via suppressing ATG7-mediated autophagy in human degenerated NPCs [41]. Additionally, p62 and LC3Ⅱ/LC3I belong to the autophagy-related proteins, of which p62 level is accumulated yet LC3Ⅱ/LC3I ratio is reduced upon the inhibition of autophagy [42,43]. In this study, we also found that the overexpression of LINC00689 raised the ATG7 level and LC3Ⅱ/LC3I value yet reduced that of p62, whereas the knockdown of LINC00689 weakened the ATG7 level and LC3Ⅱ/LC3I ratio, but elevated the p62 level in NPCs. However, ATG7 silencing abolished the effect of overexpressed LINC00689 in NPCs. These data, collectively, indicated that overexpressed LINC00689 inhibited the apoptosis of NPCs via activating ATG7-dependent canonical autophagy in NPCs, whereas LINC00689 silencing exerted the opposite effect.

Furthermore, it has already been reported that lncRNA–miRNA–mRNA network plays a critical role in IDD [9]. Zhang et al. have demonstrated that the up-regulation of lncRNA MALAT1 promoted the proliferation of NPCs and attenuated the severity of disc degeneration in IDD-modeled rats via sponging miR-503 [44]. Yang et al. have indicated that lncRNA-SLC20A1 elevated the extracellular matrix degradation in IDD NP cells via regulating the miR-31-5p/MMP3 axis [45]. In addition to that, the expression of miR-3127-5p was previously reported to be up-regulated in lumbar IDD [9]. Likewise, in our current research, we discovered that miR-3127-5p expression was up-regulated in IDD, and that LINC00689 could competitively bind with miR-3127-5p, an miRNA which could target ATG7 in NPCs. Besides, the up-regulation of miR-3127-5p reversed the effects of overexpressed LINC00689 in NPCs. These results uncovered that LINC00689 regulated the apoptosis of NPCs via miR-3127-5p/ATG7 axis-mediated autophagy.

In conclusion, in this research, we unveiled that the up-regulation of LINC00689 inhibited the apoptosis of NPCs via miR-3127-5p/ATG7 axis-mediated autophagy. These results may offer some important insights for the gene therapy of IDD.
